# Infestation of Broad Bean (*Vicia faba*) by the Green Stink Bug (*Nezara viridula*) Decreases Shoot Abscisic Acid Contents under Well-Watered and Drought Conditions

**DOI:** 10.3389/fpls.2017.00959

**Published:** 2017-06-08

**Authors:** Luisa Ederli, Cecilia Brunetti, Mauro Centritto, Stefano Colazza, Francesca Frati, Francesco Loreto, Giovanni Marino, Gianandrea Salerno, Stefania Pasqualini

**Affiliations:** ^1^Department of Chemistry, Biology and Biotechnology, University of PerugiaPerugia, Italy; ^2^Trees and Timber Institute, National Research Council of ItalySesto Fiorentino, Italy; ^3^Department of Agricultural, Food and Forest Sciences, University of PalermoPalermo, Italy; ^4^Department of Agricultural, Food and Environmental Sciences, University of PerugiaPerugia, Italy; ^5^Department of Biology, Agriculture and Food Sciences, National Research Council of ItalyRome, Italy

**Keywords:** ABA, hydrogen peroxide, *Nezara viridula*, photosynthesis, salicylic acid, *Vicia faba*, water deficit

## Abstract

The response of broad bean (*Vicia faba*) plants to water stress alone and in combination with green stink bug (*Nezara viridula*) infestation was investigated through measurement of: (1) leaf gas exchange; (2) plant hormone titres of abscisic acid (ABA) and its metabolites, and of salicylic acid (SA); and (3) hydrogen peroxide (H_2_O_2_) content. Furthermore, we evaluated the effects of experimentally water-stressed broad-bean plants on *N. viridula* performance in terms of adult host–plant preference, and nymph growth and survival. Water stress significantly reduced both photosynthesis (*A*) and stomatal conductance (*g_s_*), while infestation by the green stink bug had no effects on photosynthesis but significantly altered partitioning of ABA between roots and shoots. Leaf ABA was decreased and root ABA increased as a result of herbivore attack, under both well-watered and water-deprived conditions. Water stress significantly impacted on SA content in leaves, but not on H_2_O_2_. However, infestation of *N. viridula* greatly increased both SA and H_2_O_2_ contents in leaves and roots, which suggests that endogenous SA and H_2_O_2_ have roles in plant responses to herbivore infestation. No significant differences were seen for green stink bug choice between well-watered and water-stressed plants. However, for green stink bug nymphs, plant water stress promoted significantly lower weight increases and significantly higher mortality, which indicates that highly water-stressed host plants are less suitable for *N. viridula* infestation. In conclusion two important findings emerged: (i) association of water stress with herbivore infestation largely changes plant response in terms of phytohormone contents; but (ii) water stress does not affect the preference of the infesting insects, although their performance was impaired.

## Introduction

Climate change is increasing aridity in the Mediterranean basin (Dai, 2011; Hewitson et al., 2014), where many regions have experienced the most severe droughts on record in more recent years. Recurrent exposure to heavy and prolonged drought is dramatically increasing the vulnerability of natural ecosystems and causing massive failures in the whole agricultural sector. The imposition of water stress in plants during drought leads to inhibition of photosynthesis, which is caused by both diffusional and metabolic limitations (Centritto et al., 2003; Flexas et al., 2004). Photosynthesis is the major source of reactive oxygen species (ROS), such as hydrogen peroxide (H_2_O_2_) and singlet oxygen, even in the absence of stress. Under conditions of drought, ROS production is increased largely because of increased photorespiration, which leads to oxidative stress (Noctor et al., 2014). ROS are important secondary messengers in local and systemic signaling pathways in plants that trigger plant acclimation responses to abiotic and biotic stresses through interactions with phytohormones (Cruz de Carvalho, 2008).

Abscisic acid (ABA) is a phytohormone that is extensively involved in plant responses to abiotic stress, and especially water stress. Water stress induces accumulation of ABA in leaves and triggers downstream responses that can confer water stress tolerance to plants; e.g., stomatal closure, with the consequent feedback on photosynthesis and the ROS accumulation mentioned above. ABA-induced ROS accumulation also triggers up-regulation of the antioxidant defense systems (Jiang and Zhang, 2002). Many abiotic stresses in plants can also alter the endogenous concentrations of salicylic acid (SA), which again triggers activation of ROS-scavenging antioxidants (He et al., 2002; Erasalan et al., 2007; War et al., 2011; Sadeghi et al., 2013). For example, it has been reported that endogenous SA increases in *Phillyrea angustifolia* plants challenged with water stress, and that SA contents are positively correlated with those of α-tocopherol, which acts as a ROS and lipid-peroxyl-radical scavenger (Munné-Bosch and Peñuelas, 2003).

As plant responses to water stress might involve changes in the levels of constituents that determine the plant ‘quality’ (e.g., amino acids, organic acids, sugars, other compounds that limit negative osmotic effects), the performance and host preference of infesting herbivores might also be affected. To explain the complexity of the interactions of insect herbivores (hereafter simply called herbivores) with plants, two main hypotheses have been proposed. The plant stress hypothesis predicts that drought stress increases the hydroxylation of proteins, which subsequently increases the levels of free amino acids; this will, in turn, enhances insect growth and reproduction (White, 1984; Mattson and Haack, 1987). Alternatively, the plant vigor hypothesis suggests that vigorous (unstressed) plants will be more nutritious for herbivores (Price, 1991). Moreover, the impact of water stress on herbivores might depend on insect feeding mode (e.g., phloem feeding, chewing), or on stress occurrence over time (i.e., pulsed, continuous) (Huberty and Denno, 2004; Pineda et al., 2016). In nature, plants frequently experience simultaneous exposure to water stress and biotic stress.

Plants can respond to herbivore attacks by activating direct and indirect defenses. Direct defenses include limiting the food supply, reducing the nutrient value and insect preference, disrupting physical structures, and inhibiting chemical pathways of the insect. Indirect defenses include emission of a bouquet of volatile compounds, known as host-induced synomones, which can attract parasitoids (Kessler and Baldwin, 2001; Schoonhoven et al., 2005; Dicke, 2009; Ode, 2013). Plant synomones can be induced by insect feeding and egg deposition (Colazza et al., 2010; Hilker and Meiners, 2010; Fatouros et al., 2016). In the tritrophic system that is constituted by broad bean (*Vicia faba*)–green stink bug (*Nezara viridula* L. *Heteroptera*: *Pentatomidae*)–egg parasitoid [*Trissolcus basalis* (Wollaston) *Hymenoptera*: *Platygastridae*], the release of plant synomones is indeed induced by the combination of *N. virudula* feeding and oviposition (Colazza et al., 2004a,b). *N. viridula* is an extremely polyphagous piercing-sucking herbivore that feeds on the sap of xylem, phloem and cells, and lays egg masses mainly on the abaxial leaf surface.

Recent data from our laboratory have demonstrated that the egg parasitoid *T. basalis* shows selection toward water-stressed plants over well-watered pants (Salerno et al., 2017). However, whether water stress also influences green stink bug infestation has not been investigated to date. Moreover, the induction of direct plant defenses by green stink bug infestation remains unknown. SA, ABA, and ROS are also known to be signaling molecules in plant defenses against herbivores (Reymond, 2013), and it has been reported that SA is up-regulated and jasmonic acid (JA) is down-regulated after stink bugs have fed on soybean seeds (Giacometti et al., 2016). However, no reports are available on the involvement of such plant molecules in signaling pathways induced by green stink bug feeding and oviposition. For this purpose, H_2_O_2_ (the predominant ROS) accumulation was assessed here as an element of direct plant defenses, and SA and ABA were measured as putative components of plant signal transduction (Reymond, 2013). In addition, water stress strongly impacts on primary and secondary plant metabolism, to affect growth, health and feeding behavior of interacting herbivores (Mattson and Haack, 1987; Gutbrodt et al., 2012; Copolovici et al., 2014). Thus, we investigated whether *N. viridula* performance and preference are also affected by plant water stress. The present study was designed to test the hypothesis that water stress alters plant responses to herbivore infestation through stress-related hormones and modulation of photosynthetic parameters.

## Materials and Methods

### Plant Material

Seeds of broad-bean plants (*V. faba* cv. ‘Superaguadulce’) were immersed for 24 h in a slurry of water and non-cultivated soil (1:4) to favor root nodulation. Seeds were planted into plastic pots (9 cm × 9 cm × 13 cm) filled with a mixture of agriperlite (Superlite, Gyproc Saint-Gobain, PPC Italia, Italy), vermiculite (Silver, Gyproc Saint-Gobain, PPC Italia, Italy) and sand (1:1:1), and grown in a climate-controlled chamber with a 12-h photoperiod, photosynthetic photon flux density (*PPFD*) of 400 μmol m^-2^ s^-1^, day/night air temperatures of 24°C/20°C ± 2°C, and relative humidity from 60 to 75%. Plants were watered daily to the water capacity of the pot, and fertilized once a week with an aqueous solution of low N fertilizer with the 5:15:45 ratio of N-P-K (1.4 g L^-1^; Plantafol, Valagro, Italy).

### Insect Rearing

A colony of *N. viridula* collected in fields near Perugia (Italy) was established under controlled conditions (temperature, 24 ± 2°C; relative humidity, 70 ± 5%; light/ dark, 16 h/8 h) in plastic cages (50 cm × 30 cm × 35 cm), with mesh-covered holes for ventilation (diameter, 5 cm). The insects were fed regularly with a diet of sunflower seeds and seasonal fresh vegetables.

### Water Stress and Herbivore Treatment

The water stress treatment was applied to 15-day-old plants with approximately four fully expanded leaves, and two independent experiments were performed. On the day prior to water stress onset, the plants were fully irrigated and the excess water was allowed to drain off overnight. After draining, the pots were weighed to 1.0 g precision on a digital balance (model QS32A; Sartorius Instrumentation, Ltd, Germany), to determine the pot weight (PW) at the pot maximum water capacity (*Initial*_PW_). The pots were wrapped in plastic bags to prevent evaporation from the soil, and weighed daily during the experiment to determine the daily pot weight (*Daily*_PW_). Progressive soil water deprivation was expressed as the fraction of transpirable soil water (FTSW) (Sinclair and Ludlow, 1986; Brilli et al., 2013). The water stress cycle ended when transpiration of the stressed plants decreased to ∼10% of the mean transpiration of the control plants, which was achieved 12 days after withholding water. The pots were then weighed to determine the final weight (*Final_PW_*) of the water-stressed plants. The FTSW was then calculated as in Equation (1):

(1) $$\text{FTSW}\;=\;(Daily_{\text{PW}}-Final_{\text{PW}})/(Initial_{\text{PW}}-Final_{\text{PW}}).$$

During the 12 days of water deprivation, the plants were grown to the seven fully expanded leaf. Well-watered plants were used as controls: four plants at the beginning of the treatment (i.e., control), and four plants at the end of the 12 days of water deprivation (i.e., developmental control). Biochemical analyses and gas exchange measurements were performed on both the control and developmental control plants. The developmental control measurements taken at the end of the experiments showed no significant variations with respect to control plants (data not shown).

Each of four plants for treatment as control (FTSW_100_) and water-stressed (FTSW_80_, FTSW_50_, FTSW_10_) were exposed to three gravid *N. viridula* females for 24 h. During this period, the insects walked freely, fed and oviposited on the whole plants. Twenty-four hours after insect removal, the leaves with egg masses were used for biochemical analyses and gas exchange measurements.

### Gas Exchange and Chlorophyll Fluorescence Measurements

Gas exchange and chlorophyll fluorescence measurements were performed on four plants per water treatment, using a portable infrared gas analyser equipped with an integrated fluorometer (LI-6400; Li-Cor, Inc., Lincolin, NE, United States), with a 2 cm^2^ leaf chamber. Measurements were performed on the central portion of the first four fully expanded leaves of the plants, at FTSW_100_, FTSW_80_, FTSW_50_, and FTSW_10_. The measurements were made at the CO_2_ concentration of 390 μmol mol^-1^, relative humidity of 35–45%, and leaf temperature of 25°C. An outer gasket was added to the LI-6400 leaf clamp, to create a buffer zone in which the H_2_O and CO_2_ gradients between the in-chamber air and the pre-chamber air were minimized by the supply of the infrared gas analyser exhaust air (Rodeghiero et al., 2007). Photosynthesis (*A*), stomatal conductance (*g*_s_) and chlorophyll fluorescence were measured simultaneously at PPFD = 800 μmol m^-2^ s^-1^. After determining steady-state fluorescence (*F_s_*), the maximum fluorescence (*F*_m_′) was measured by applying a saturating light pulse (10,000 μmol m^-2^ s^-1^; 0.8 s duration), and the minimal level of fluorescence (*F_0_*′) was measured by switching off the actinic light after the saturating pulse. The photosystem-II operating photochemical efficiency (*Φ*_PSII_) was calculated according to Equation (2) (Genty et al., 1989):

(2) $$\Phi_{\text{PSII}}\;=\;(F_{\text{m}}{}^{\prime }-F_{s})/F_{\text{m}}{}^{\prime }.$$

The photochemical quenching (*qP*), related to the proportion of open PSII reaction centeres was determined according to Equation (3) (Schreiber, 1986):

(3) $$qP\;=\;(F_{\text{m}}{}^{\prime }-F_{s})/(F_{\text{m}}{}^{\prime }-F_{0}{}^{\prime }).$$

Finally, the electron transport rate (ETR) was calculated according to Equation (4):

(4) $$ETR\;=\;\Phi_{\text{PSII}}\;\times \;PPFD\;\times \;0.5\;\times \;0.87,$$

where the coefficient 0.5 assumes equal distribution of the absorbed photons between PSI and PSII, and 0.87 is the leaf absorbance coefficient (Krall and Edwards, 1992). The measurements were conducted on a total of eight replicates.

### Hormones and H_2_O_2_ Quantification

Biochemical analyses were performed on leaf and root samples collected from four different non-infested and infested plants subjected to the FTSW treatment. In non-infested plants, the four leaves (from the first to the fourth completely expanded leaves) and root apical portions of each single plant were sampled. For the biochemical analyses of the plants infested with green stink bug, leaves with eggs (with the exception of cotyledons and leaves that were not completely expanded) and roots were collected. The leaf and root samples were rapidly frozen by immersion in liquid nitrogen.

Total SA was extracted and quantified as reported by Di Baccio et al. (2012). For quantification of ABA and its metabolites, fresh leaf tissue (300–350 mg) was added to 50 ng deuterated ABA (d^6^-ABA), 50 ng d^5^-ABA-glucose ester (d^5^-ABA-GE), 50 ng d^3^-phaseic acid (d^3^-PA), and 50 ng d^3^-dihydrophaseic acid (d^3^-DPA). The sample was extracted with 3 mL CH_3_OH/H_2_O (1:1 [v/v], pH 2.5 with HCOOH) at 4°C for 30 min. The supernatant was partitioned with 3 mL × 3 mL *n*-hexane, and the aqueous- methanolic phase was loaded onto C18 cartridges (Sep-Pak; Waters, Milford, MA, United States), and washed with 2 mL water, pH 2.5. Free-ABA and ABA-GE were then eluted with 1.2 mL ethylacetate, and the eluate was dried under nitrogen, and rinsed with 500 μL CH_3_OH/H_2_O (1:1 [v/v], pH 2.5 with HCOOH). Identification and quantification of free-ABA and ABA metabolites were performed using liquid chromatography–electrospray ionization-tandem mass spectrometry. The samples (3 μL) were injected into the chromatograph (LC1200; Agilent Technologies, Santa Clara, CA, United States), which was coupled to a triple quadrupole mass spectrometer detector equipped with an electrospray ionization source (6410; Agilent Technologies) operating in negative ion mode. The metabolites were separated in a C_18_ column (Poroshell; 3.0 mm × 100 mm, 2.7 μm i.d.; Agilent Technologies) at a flow-rate of 0.3 mL min^-1^ and using a linear gradient solvent system from 95% solvent A (0.1% HCOOH in H_2_O) to 100% solvent B (CH_3_CN/MeOH, 1:1 [v/v], with 0.1% HCOOH), over 30 min. Quantification was conducted in multiple reaction mode, as reported by López-Carbonell et al. (2009).

For determination of H_2_O_2_, fresh plant tissue (500 mg) was ground to a powder in liquid nitrogen and homogenized in 2 mL 0.2 M HClO_4_ in a pre-cooled pestle and mortar. The extract was centrifuged at 10,000 × *g* for 10 min at 4°C. The acidic supernatant was neutralized to pH 6.5–7.0 with 0.2 M NaOH, and centrifuged at 3,000 × *g* for 2 min, to sediment insoluble material. To remove pigments, antioxidants, polyphenolics and other interfering substances, activated charcoal was added, and the extract was centrifuged at 11,000 × *g* for 5 min at 4°C. The supernatant was filtered through PTFE membranes (0.45 μm; Millipore), and the H_2_O_2_ concentrations were immediately determined using the xylenol orange assay, as reported previously (Pasqualini et al., 2003), with the calculations using a standard curve prepared with known concentrations of H_2_O_2_. Biochemical data were expressed on a dry weight basis by weighing parallel samples of fresh plant tissues (FW) and drying them at 70°C for at least 72 h to determine the dry weight (DW). The specific leaf water content (SWC), as g H_2_O g^-1^ DW, was calculated according to the following formula:

(5) SWC = (FW - DW)/DW.

### *Nezara viridula* Host Preference and Performance

*Nezara viridula* females had the choice of where to lay their eggs, in terms of FTSW_100_ and water-stressed (FTSW_80_, FTSW_50_, or FTSW_10_) broad-bean plants. One control and one water-stressed plant were placed together inside a glass box (36 cm × 34 cm × 50 cm) with the above-ground parts isolated from the pots using a plastic panel. Fifteen gravid *N. viridula* females were released into the box and allowed to move freely under controlled conditions (temperature, 26 ± 1°C; relative humidity, 30 ± 5%; light/dark, 12 h/12 h). After 24 h, the number of females on each plant and the number of egg masses deposited per plant were counted. In total, 10 pairwise comparisons between FTSW_100_ and each of FTSW_80_, FTSW_50_, and FTSW_10_ were carried out. The percentages of adult choice and egg masses deposited were recorded. To determine whether these three levels of water stress affected the herbivore growth, *N. viridula* nymphs were placed on FTSW_100_ and FTSW_80_, FTSW_50_, and FTSW_10_ plants. After placing the above-ground organs inside a tissue bag, each plant was inoculated with 10 nymphs (3rd instar) that had been weighed previously on a microbalance (P Series; Exacta, Germany). The nymphs were allowed to feed on the individual plants for 1 week under controlled conditions (temperature, 26 ± 1°C; relative humidity, 30 ± 5%; light/dark 15 h/9 h). To maintain a constant FTSW during the entire bioassay period, the transpired water was returned to the pots every 2 days. At the end of the experiment, the following were recorded: nymph weight increases (i.e., % difference between final and initial weights in relation to the initial weight), and nymph mortality (i.e., % dead nymphs in relation to total nymphs placed on each plant at the beginning of the experiment). Moreover, the live nymphs were weighed and the mean weight for each plant was calculated to record the nymph weight increase (i.e., % difference between final and initial weights in relation to initial weight). This bioassay was performed with 10–13 individual plants per water stress level.

### Statistical Analysis

The data for gas exchange and biochemical analyses underwent factorial analysis of variance (ANOVA; two-way maximum interactions) to determine the effects of the interactions between water stress and insect infestation on all of the dependent variables. *Post hoc* multiple comparisons were carried out using Tukey honest significant difference tests, to analyze the differences among the treatment means for the physiological and biochemical data. *N*. *viridula* choice and oviposition were compared statistically by parametric paired *t*-tests for dependent samples. Nymph weight increase (%) and nymph mortality (%) were analyzed using one-way factorial ANOVA. For *post hoc* comparisons, Dunnett tests were used to compare water-stressed and control plants (Statsoft Inc., 2001). Before analysis, Box–Cox transformations were used to reduce the data heteroscedasticity (Sokal and Rohlf, 1998).

## Results

### Gas Exchange and Chlorophyll Fluorescence Measurements

**FIGURE 1:**
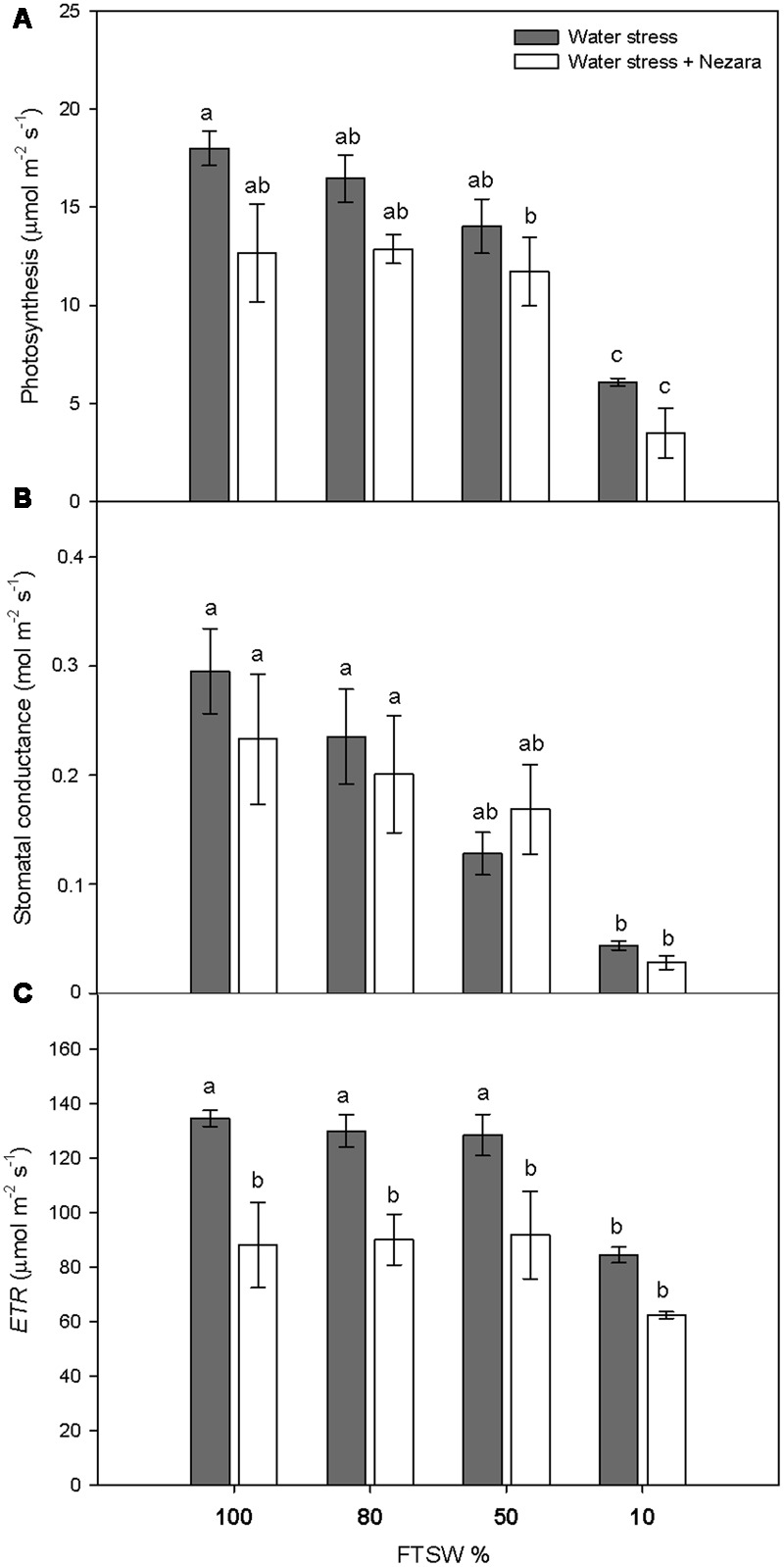
Gas exchange measurements in well-watered and water-stressed broad bean plants with or without *Nezara viridula* infestation. Leaf photosynthesis **(A)**, stomatal conductance **(B)**, and electron transport rate (*ETR*) **(C)**, of *Vicia faba* plants under developing water stress (gray bars) and with an associated presence of *N. viridula* infestation (white bars). Measurements were made at 100% (well-watered plants), and 80, 50, and 10% of fraction of transpirable soil water (FTSW). Data are means ± SE (*n* = 8). Different letters indicate significant differences (*P* < 0.05).

Under well-watered conditions (FTSW_100_), the SWC was 13.51 ± 1.44 g H_2_O g^-1^ DW. During the progression of water stress, SWC did not change significantly for mild (FTSW_80_) and moderate (FTSW_50_) water stress. Under severe water stress (FTSW_10_), SWC dropped significantly to 7.85 ± 0.13 g H_2_O g^-1^ DW (*P* ≤ 0.001). Photosynthesis (*A*) (**Figure 1A**) and stomatal conductance (*g*_s_) (**Figure 1B**) of *V. faba* plants significantly decreased with increasing water stress, both in the absence and presence of *N. viridula*. The FTSW thresholds at which *A* and *g*_s_ began to decline rapidly where different, as *g*_s_ declined at a higher rate with respect to *A*, and the difference with respect to well-watered plants was significantly different already at FTSW_50_ (**Figure 1B**). Under severe water stress conditions (FTSW_10_), the *A* and *g_s_* reductions compared to well-watered leaves were around 65 and 85%, respectively. The response of the ETR to soil drying (**Figure 1C**, *ETR*) mirrored that of *A*, with a decrease of about 37% at FTSW_10_, from the pre-stress values. *N. viridula* infestation had small, and non-significant, effects on the responses of *A* and *g*_s_ to water stress. In contrast, *ETR* significantly decreased in plants infested by *N. viridula*, by 35% (at FTSW_100_) and 26% (at FTSW_10_), with respect to non-infested plants at comparable FTSW.

### ABA Metabolism

**FIGURE 2:**
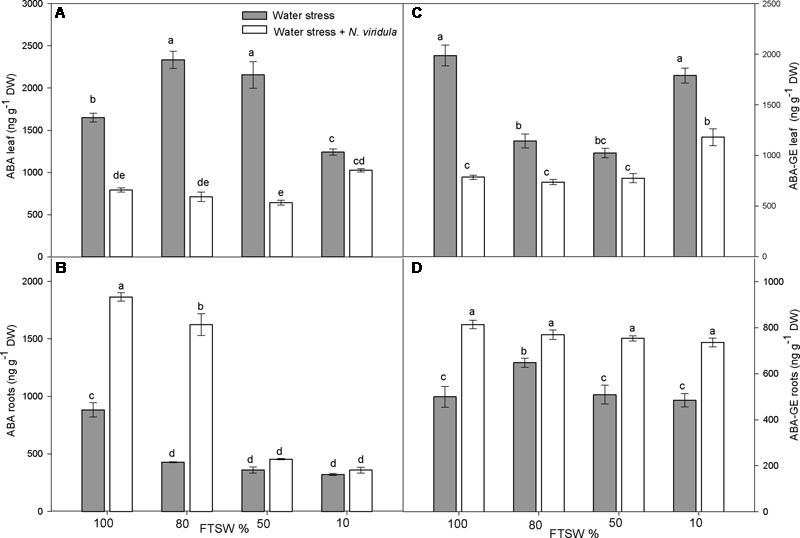
Quantitative measurements (ng g^-1^ DW) of ABA and its metabolite ABA-GE in well-watered and water-stressed broad bean plants with or without *N. viridula* infestation. Abscisic acid (ABA; **A,B**) and abscisic acid glucose ester (ABA-GE; **C,D**) contents in leaves **(A,C)** and roots **(B,D)** of *V. faba* plants under developing water stress (gray bars) and with an associated presence of *N. viridula* infestation (white bars). Measurements were made at 100, 80, 50, and 10% of FTSW. Data are means ± SE (*n* = 8). Different letters indicate significant differences (*P* < 0.05).

**FIGURE 3:**
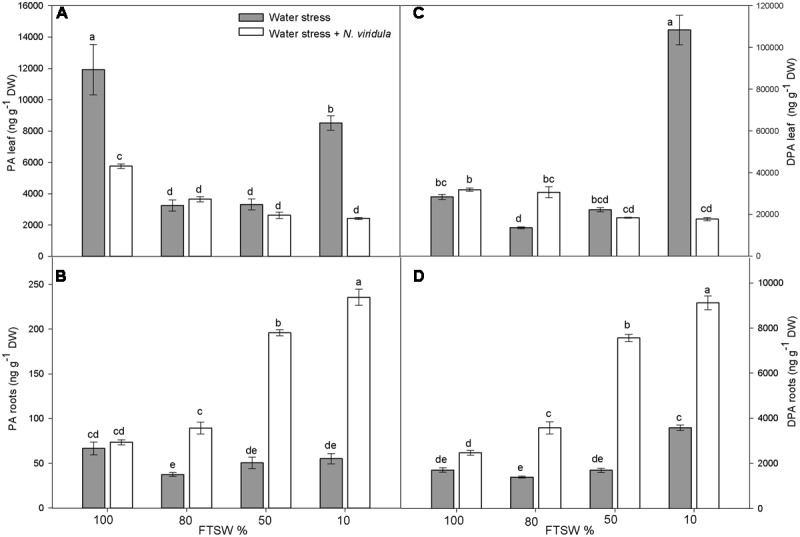
Quantitative measurements (ng g^-1^ DW) of ABA catabolites PA and DPA in well-watered and water-stressed broad bean plants with or without *N. viridula* infestation. Phaseic acid (PA; **A,B**) and dihydrophaseic acid (DPA; **C,D**) in leaves **(A,C)** and roots **(B,D)** of *V. faba* plants under developing water stress (gray bars) and with an associated presence of *N. viridula* infestation (white bars). Measurements were made at 100, 80, 50, and 10% of FTSW. Data are means ± SE (*n* = 8). Different letters indicate significant differences (*P* < 0.05).

The leaf and root ABA and ABA-GE contents (**Figure 2**) and the content of the ABA catabolites phaseic acid (PA) and dihydrophaseic acid (DPA) (**Figure 3**) were analyzed in plants under water stress and for the interaction between water stress and *N. viridula* infestation.

Along the water stress treatments, free ABA content in the leaves increased significantly at FTSW_80_ and FTSW_50_, and decreased significantly at FTSW_10_, compared to well-watered plants (**Figure 2A**). In roots, a significant decrease in free ABA content was observed at each stage of water stress (**Figure 2B**). The infestation with *N. viridula* caused contrasting responses on leaf and root contents of free ABA in plants before and during water stress. In leaves of infested plants, the free ABA content was significantly lower than in non-infested plants at FTSW_100_, FTSW_80_ and FTSW_50_, whereas there was no difference between treatments at FTSW_10_ (**Figure 2A**). In roots, the content of free ABA at FTSW_100_ was more than double for the infested compared to non-infested plants. Under mild water stress (FTSW_80_) this difference was even higher. However, as the water stress became more severe (FTSW_50_, FTSW_10_), the root free ABA content dropped in plants infested by *N. viridula* (**Figure 2B**). Leaf ABA-GE contents (**Figure 2C**) showed generally opposite trends to those of root free ABA in response to water stress, with significant decreases at FTSW_80_ and FTSW_50_ (by ∼40%), while returning to pre-stress levels at FTSW_10_. In the roots, there was no clear pattern of ABA-GE in response to water stress, as ABA-GE only significantly increased (by ∼40%) at FTSW_80_. *N. viridula* infestation induced a significant further reduction in leaf ABA-GE, especially in well-watered leaves, but also at different water stress levels. However, when comparing only plants infested by *N. viridula*, the ABA-GE content was significantly higher at FTSW_10_ than at any other FTSW (**Figure 2C**). In the roots of plants infested by *N. viridula*, the ABA-GE contents increased significantly compared to non-infested plants at each water stress level, and was similarly high across all FTSW (**Figure 2D**).

Water stress significantly reduced the leaf content of PA, by 73% at FTSW_80_ and FTSW_50_, but by only 29% at FTSW_10_ (**Figure 3A**). The PA content also decreased in the roots in response to the water stress, although it was significantly lower than in well-watered plants (by ∼35%) only at FTSW_80_ (**Figure 3B**). *N*. *viridula* infestation significantly decreased the leaf PA content of well-watered plants (by ∼60%) (**Figure 3A**), but did not affect its content in roots (**Figure 3B**). Furthermore, *N*. *viridula* infestation also modified the response to water stress. The leaf PA content showed a declining trend as water stress increased in severity (significant only at FTSW_10_). An opposite, significantly increasing trend occurred in water-stressed roots, which resulted in about a threefold increase in the PA content at FTSW_10_. The DPA content was also affected by water stress, although significant increases were only seen at FTSW_10_ for both leaves (by 388%) (**Figure 3C**) and roots (by 110%) (**Figure 3D**). No effects of *N*. *viridula* infestation on DPA contents were evident at FTSW_100_ for both leaves and roots. However, similar to PA, as the water stress increased in severity, the DPA contents showed a declining trend in leaves and an opposite significant increasing trend in roots. At FTSW_10_, the root DPA content increased by more than fourfold after *N*. *viridula* infestation (**Figure 3D**).

### H_2_O_2_ and SA

**FIGURE 4:**
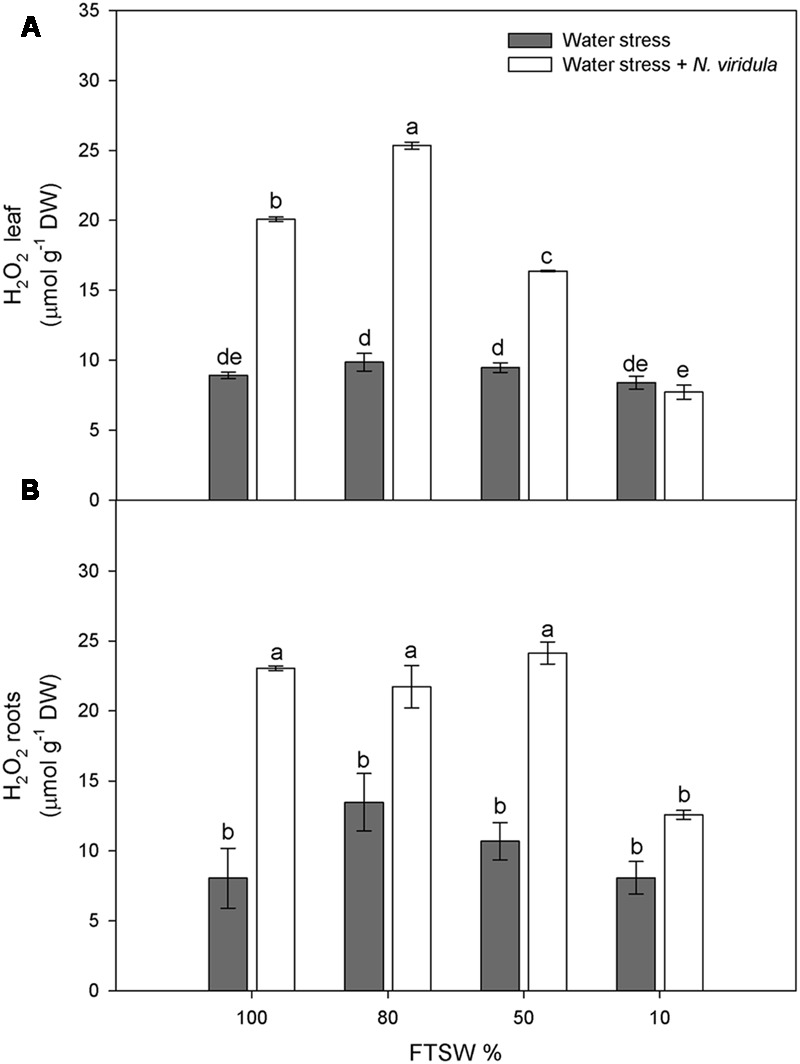
Quantitative measurements (μmol g^-1^ DW) of H_2_O_2_ in well-watered and water-stressed broad bean plants with or without *N. viridula* infestation. Quantitative measurements of H_2_O_2_ in leaves **(A)** and roots **(B)** of *V. faba* plants under developing water stress (gray bars) and with an associated presence of *N. viridula* infestation (white bars). Measurements were made at 100, 80, 50, and 10% of FTSW. Data are means ± SE (*n* = 8). Different letters indicate significant differences (*P* < 0.01).

**FIGURE 5:**
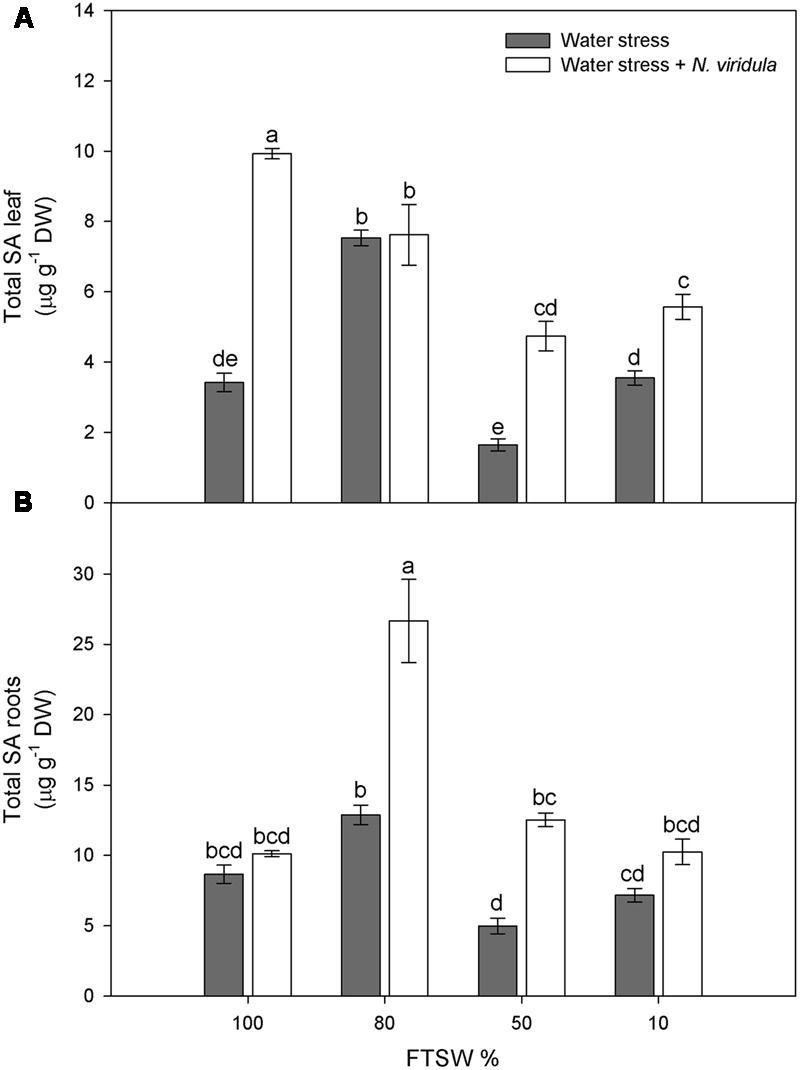
Quantitative measurements (μg g^-1^ DW) of total SA in well-watered and water-stressed broad bean plants with or without *N. viridula* infestation. Total salicylic acid (SA) contents in leaves **(A)** and roots **(B)** of *V. faba* plants under developing water stress (gray bars) and with an associated presence of *N. viridula* infestation (white bars). Measurements were made at 100, 80, 50, and 10% of FTSW. Data are means ± SE (*n* = 8). Different letters indicate significant differences (*P* < 0.01).

Water stress did not significantly affect the H_2_O_2_ contents of non-infested plants for either leaves (**Figure 4A**) or roots (**Figure 4B**). However, *N*. *viridula* infestation significantly stimulated the production of H_2_O_2_ in well-watered and water-stressed leaves, until the stress reached FTSW_10_ (**Figure 4A**). A similar trend was observed in infested plant roots (**Figure 4B**). SA showed similar trends in response to water stress in non-infested leaves (**Figure 5A**) and roots (**Figure 5B**), peaking under mild water stress (FTSW_80_), and then dropping again when the water stress further increased. The infestation of *N. viridula* caused a very large increase in SA content in well-watered leaves and in roots at FTSW_80_. The amount of SA was also higher in infested than in non-infested leaves and roots at FTSW_50_.

### *Nezara viridula* Host Preference and Performance

**FIGURE 6:**
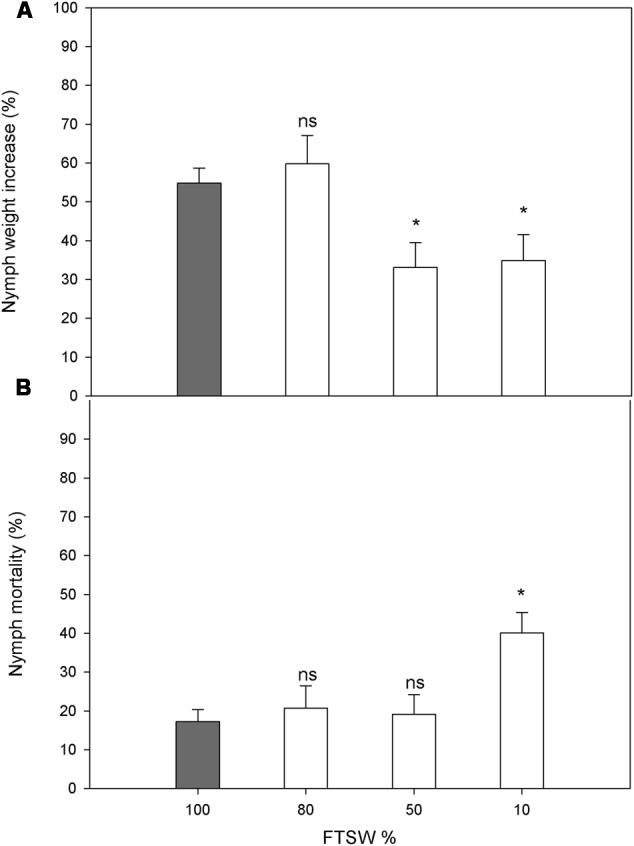
Performance of *N. viridula* nymphs in well-watered and water-stressed plants. Weight increase **(A)** and mortality **(B)** of *N. viridula* nymphs feeding on *V. faba* plants under developing water stress. Measurements were made at 100% (*n* = 11), 80% (*n* = 10), 50% (*n* = 13), and 10% (*n* = 11) of FTSW. Data are means ± SE. White bars are compared with gray bar (well-watered), ^∗^*P* < 0.05; ns, no significant difference.

The water stress did not affect the choice of host plant by the female *N. viridula* (FTSW_80_, 50.1 ± 4.9%; FTSW_50_, 51.5 ± 5.4%; FTSW_10_, 49.9 ± 5.0%), and similarly, no differences were seen in egg-mass distribution between well-watered and water-stressed plants (FTSW_80_, 52.9 ± 10.9%; FTSW_50_, 52.1 ± 9.7%; FTSW_10_, 49.6 ± 7.8%). The nymph weight increase was not affected in plants under mild water stress (FTSW_80_), but was significantly reduced for FTSW_50_ and FTSW_10_ (**Figure 6A**). A significant increase in the mortality of *N. viridula* nymphs was only seen in response to severe water stress (FTSW_10_) (**Figure 6B**).

## Discussion

As climate change continues, droughts and insect populations are predicted to increase in many parts of the world (Oliver and Morecroft, 2014). It is therefore important to understand how such changes can affect the agroecosystems. The present study suggests that in a typical legume crop in temperate areas: (i) association of water stress with herbivore infestation greatly changes the plant responses in terms of phytohormone contents; although (ii) water stress does not change the plant preference of the infested insects, although it does greatly impair the nymph vitality after feeding on leaves.

As expected, water stress induced a significant decrease in the photosynthetic parameters. In several studies where water stress was expressed as a function of FTSW, *A* and *g*_s_ were reduced in parallel; e.g., for poplar (Brilli et al., 2007; Centritto et al., 2011) and eucalyptus (Brilli et al., 2013). In the present study, with respect to *A* (Brilli et al., 2007), *g*_s_ showed slightly higher curve inflection, from where it began to decline rapidly (i.e., the threshold at which the second stage of the plant responses to FTSW started; Sinclair and Ludlow, 1986). This indicates that *A* is primarily influenced by CO_2_ diffusive limitations when water stress is not severe (i.e., FTSW_80_ and FTSW_50_ in the present study) (Cornic, 2000; Lawlor and Cornic, 2002; Centritto et al., 2003; Brilli et al., 2007; Flexas et al., 2013). Generally, the combined effects of biochemical and diffusional factors appear to limit *A* only under severe water stress (FTSW_10_) (Lawlor and Cornic, 2002), whereas *N. viridula* infestation had a limited negative effect on *A* and *g*_s_ of well-watered and water-stressed leaves. However, the infestation significantly reduced *ETR* across all FTSW levels, which implies damage to the photochemistry of photosynthesis during insect feeding, which will probably be related to damage to photosynthetic pigments. Herbivory effects on photosynthetic parameters are controversial and might be different according to the insect feeding behaviors. Chewing insects can cause extensive damage to plant tissues, whereas piercing-sucking insects (such as *N*. *viridula*) generally induce minimal physical damage (War et al., 2013). However, previous studies on plant injury caused by insects with piercing–sucking mouthparts have also shown that *A* and *g*_s_ can decrease or increase as a result of herbivore feeding (Meyer and Whitlow, 1992; Kerchev et al., 2012). Studies that have shown increases or maintenance of the photosynthetic activity indicate a compensatory response of the plants; i.e., increased *A* of non-infested leaf parts (Crawley, 1989; Welter, 1989). Maintenance of *A* might also indicate an increased allocation of energy and carbon-based resources to the plant defensive systems (Schwachtje and Baldwin, 2008). Indeed, tolerant lines of barley and wheat can be discriminated from susceptible lines on the basis of their maintenance of *A* under herbivore infestation (Franzen et al., 2007; Gutsche et al., 2009).

Water stress induced changes in the contents of ABA and its catabolites in both leaves and roots of *V*. *faba*. The increased free ABA contents in leaves at mild to moderate water stress (FTSW_80_, FTSW_50_) were correlated to low levels of its oxidized catabolites, and to increased hydrolysis of its conjugate form, ABA-GE. Increased free ABA content might be the cause for the *g*_s_ reduction as the water stress increases (Davies and Zhang, 1991). It should be noted, however, that free ABA content increased even in leaves under mild water stress (FTSW_80_) with no significant reduction of *g*_s_ seen. This early increase in leaf free ABA might be associated to the concurrent decrease in root free ABA, because ABA is transported to the aerial part as a mechanism for the transmission of a chemical signal to indicate the declining soil water status (Sauter et al., 2001; Zhang et al., 2006). Moreover, the relationship between free ABA and *g*_s_ was lost under severe water stress (FTSW_10_), when the catabolism of ABA increased and the physiological functions of the plants were compromised, possibly because of reduced xylem flow (Atkinson et al., 2015) and a further drop in leaf water potential (Brodribb and McAdam, 2013). Low xylem flow might also explain the stable root ABA content even under severe water stress.

The trends seen for the leaf ABA-GE contents were specular of those of free ABA at all stages of stress, which suggests a ‘buffer’ function for this physiologically inactive form of free ABA (Jiang and Hartung, 2008). ABA-GE can be cleaved by a dehydration-inducible β-glucosidase that contributes to the regulation of free ABA content in leaves (Sauter et al., 2002; Lee et al., 2006; Xu et al., 2012). In the roots, the ABA-GE content remained constant during the water stress, and did not appear to be involved in fine control of the free ABA content. Conversely, in the plants infested by *N. viridula*, the free ABA content showed very large changes. The main trends were a reduction of leaf free ABA at all water contents, and an increase in root free ABA in the controls (FTSW_100_) and under mild water stress (FTSW_80_) conditions. These trends were confirmed by the levels of the ABA catabolites, PA and DPA, which remained low in leaves during stress progression, whereas they increased significantly in roots. It is known that ABA influences JA biosynthesis and JA-dependent gene expression, thereby activating resistance to herbivores (Adie et al., 2007; Bodenhausen and Reymond, 2007; Vos et al., 2013). The down-regulation of ABA synthesis and signaling in leaves can be interpreted as part of the *N. viridula* attack strategy to inhibit JA synthesis and JA-dependent defense gene expression, and therefore keep the defenses of the host plant low.

The importance of oxidative stress in plant water stress responses is widely acknowledged (Smirnoff, 1993; Cruz de Carvalho, 2008; Miller et al., 2010). This can range from oxidative damage to the role of ROS in local and systemic signaling. Increased ROS production under water stress has been associated with inhibition of photosynthesis. Stomatal closure induced by water stress restricts CO_2_ uptake, which in turn favors photorespiratory production of H_2_O_2_ in the peroxisomes, and the production of superoxide, H_2_O_2_ and singlet oxygen by the photosynthetic electron transport chain (for review, see Noctor et al., 2014). Furthermore, H_2_O_2_ enhancement under stress might induce acclimation/defense responses triggered by ABA-mediated stomatal closure (Pei et al., 2000).

However, many studies have shown no changes in H_2_O_2_ contents in response to water stress (Moran et al., 1994; Porcel and Ruiz-Lozano, 2004). This is also the case for the present study, as the H_2_O_2_ contents of both leaves and roots were not affected by the water stress, despite stomatal closure and the consequent inhibition of photosynthesis. Activation of the ROS scavenging enzymes is under the control of SA, which promotes plant tolerance to abiotic stress (He et al., 2002; Erasalan et al., 2007; War et al., 2011; Sadeghi et al., 2013). The significant increase in leaf SA content under mild water stress (FTSW_80_) might be the signal that promotes the induction of antioxidant enzymes that can counteract H_2_O_2_ accumulation under water stress, and under combined water stress and *N. viridula* infestation. Interestingly, the highest stimulation of leaf SA after infestation with *N. viridula* was observed here in the well-watered plants. Thus, while both abiotic and biotic stresses can induce SA biosynthesis, there were no additive effects under the co-occurring stress. Many studies have shown that in tobacco and cucumber leaves inoculated with necrotising pathogens, there is export of SA into the phloem (Métraux et al., 1990; Rasmussen et al., 1991; Yalpani et al., 1991; Shulaev et al., 1995). Similarly, the absence of an additive increase in SA in these infested and water-stressed *V. faba* leaves might be due to partial reallocation of the SA toward the roots, where SA indeed showed a large increase. The observation that both H_2_O_2_ and SA accumulate in leaves where a green stink bug has laid at least one batch of eggs suggests that oviposition causes a localized response with a strong similarity to a hypersensitive response (Little et al., 2007; Bruessow et al., 2010). It remains to be clarified whether this response to egg deposition constitutes a direct defense or a mechanism to anticipate the threat posed by the future feeding of the larvae.

The final focus of this study was to evaluate how water stress of *V. faba* influences pest infestation and nymph growth performance. The highly debated relationships between plant stress, plant quality, and herbivore performance has generated several hypotheses (White, 1984; Price, 1991; Huberty and Denno, 2004). Under the experimental conditions also used in the present study, water stress induction of plant volatiles attracted more individuals of the egg parasitoid *T. basalis* (Salerno et al., 2017). However, here *N. viridula* females showed no preference in terms of where they laid their eggs according to the well-watered and water-stressed *V. faba* plants. The neutral behavioral choice of *N. viridula* toward the water-stressed plants did not correspond to the performance of the herbivore larvae, which was significantly lowered by the water stress. Thus, the water-stressed hosts were less suitable for the *N. viridula*. Reductions in lepidopteran larvae performance and aphid population densities due to water stress in host plants have been reported (Huberty and Denno, 2004; Badenes-Perez et al., 2005; Nachappa et al., 2016). This might be due to reduced biosynthesis of the primary metabolites (e.g., carbohydrates, proteins) caused by increased limitation of photosynthesis or early plant senescence under water stress, or by insufficient water availability in the drying plant tissues (Showler, 2013). Indeed, these effects were observed in the present study when the water stress was already relatively severe, as the photosynthetic activity and SWC dropped significantly. The negative effects of water stress on insect growth and survival might also be due to accumulation of ‘anti-nutrients.’ A negative effect on egg production of the SA analog benzo (1,2,3) thiadiazole-7-carbothioic acid *S*-methyl ester was reported for the herbivore mite *Tetranychus urticae* (Choh et al., 2004). Also, as SA is a metabolite that can accumulate in leaves in response to water stress, it might have been involved in reductions in the nutritional properties of the leaves or the feeding capacity of the insects. However, this hypothesis must be tested in further experiments.

Overall, our data show that water stress and *N. viridula* infestation individually trigger the SA pathway. However, the water stress does not affect the H_2_O_2_ contents, which were only increased by the *N. viridula* infestation. In contrast, ABA signaling has a role under water stress, and is down-regulated by *N. viridula* infestation. Furthermore, under our experimental conditions, the decrease in *N. viridula* performance suggests that this herbivore is adversely affected by severe water stress. These findings provide a better understanding of how plants respond to combined abiotic and biotic stresses.

## Author Contributions

All of the authors participated in the conception and design of the experiments; LE, GS, FF, GM, and CB performed the experiments and analyzed the data; SP, LE, MC, and FL wrote the manuscript; and all authors interpreted the results and revised the manuscript.

## Conflict of Interest Statement

The authors declare that the research was conducted in the absence of any commercial or financial relationships that could be construed as a potential conflict of interest.
